# Reasons for Discontinuation or Termination of Help‐Seeking for Psychosocial Support Services Among Patients With Cancer in Japan

**DOI:** 10.1002/pon.70642

**Published:** 2026-11-12

**Authors:** Tomoko Matsui, Kanako Taku

**Affiliations:** ^1^ Graduate School of Human Sciences The University of Osaka Suita Osaka Japan; ^2^ Department of Psychology Oakland University Rochester Michigan USA

**Keywords:** cancer, help‐seeking behavior, mental health, oncology, patients with cancer, psychosocial support services, service discontinuation, service termination

## Abstract

**Objective:**

Studies on help‐seeking behavior for psychosocial support services among patients with cancer have mostly focused on the initiation of service use. This study aimed to describe the reasons why the patients decided to discontinue or terminate psychosocial support services and examine the relationships between service needs and psychological distress, as well as cancer‐related worries.

**Methods:**

A cross‐sectional web‐based survey was conducted on patients with cancer. Participants provided their demographic characteristics, details regarding the use of psychosocial support services, reasons for discontinuation/termination of such services, perceived need for continued use, psychological distress, and cancer‐related worries.

**Results:**

Among 510 respondents, 28 (5.5%) reported discontinuing or terminating psychosocial support services. The most frequently reported reasons reflected need‐fulfilling processes, such as “problems were resolved” and “satisfied.” Service mismatch and structural/access barriers were also mentioned. Additionally, one‐way analyses of variance showed that those who wished to continue using the services reported more worries about physical and symptomatic problems compared with those who did not (*η* ^2^ = 0.127).

**Conclusions:**

Discontinuation or termination of psychosocial support services should not be uniformly interpreted as dropout or service failure, as it may occur due to various reasons, including positive changes such as problems being resolved or coping resources being enhanced, or changes in priorities and needs. Patients who reported that they would have preferred to continue using psychosocial support services tended to report higher levels of physical and symptomatic problems, underscoring the need for ongoing assessment and flexible pathways back into care.

## 1 Background

Patients with cancer often experience cancer‐related problems and distress after diagnosis, including psychological, physical, and social difficulties [1]; therefore, psychosocial support services (PSS) are provided to alleviate and manage them. However, many patients tend not to use PSS, regardless of the extent to which they recognize their distress [2, 3, 4]. The use of PSS can be understood within the framework of help‐seeking behavior (HSB), a complex decision‐making process aimed at problem solving that involves planning and interpersonal interactions with specific professionals [5]. Previous studies in cancer care settings revealed support needs, identified facilitators and barriers to service use, and applied behavior‐change models to explain the usage of PSS [2, 6, 7, 8, 9, 10].

Identifying the reasons why patients discontinued or terminated using PSS is not straightforward. The discontinuation or termination of PSS is a multifaceted phenomenon that includes both negative and positive aspects. For example, worries or unresolved issues may remain even after termination, and early termination due to satisfaction may sometimes be interpreted as “dropout.” [11, 12, 13] Studies have revealed that patients discontinue or terminate using PSS for various reasons, including intervention‐related barriers (e.g., dissatisfaction, program complexity and burden, and patient–provider relationship issues), environmental barriers (e.g., lack of time, difficulties in access, and financial constraints), adherence to cancer treatment, and physical conditions such as disease worsening [11, 13]. Moreover, some patients denied that they “dropped out” or were confused about being labeled as dropouts, perhaps because they often did not mean to. Several studies also reported reasons for discontinuation as “unknown.”

Investigating the reasons why patients with cancer initiated, continued, discontinued, or terminated PSS use should provide a broader understanding of HSB. Existing research on PSS discontinuation/termination has mainly focused on limited populations and contexts. Recently, a wide range of PSS options have become available. The current study focused on the post‐initiation phase of HSB for diverse PSS among patients with cancer in Japan. We aimed to describe reasons for discontinuation or termination of service use and examine the relationship with willingness to continue services and psychological distress, as well as cancer‐related worries. HSB may be influenced by cultural and healthcare system contexts [14]. Therefore, examining HSB among patients with cancer in Japan, where PSS are widely available yet underutilized, may offer insights transferable to similar healthcare systems and cultural contexts.

## 2 Methods

### 2.1 Procedure and Participants

A cross‐sectional web‐based survey was conducted by INTAGE Inc. in Japan, between March 25 and 29 in 2022. Sample included (1) patients with cancer who were not hospitalized for a long period and (2) patients who were diagnosed cancer within the past 5 years. An invitation to participate in the screening survey was distributed to members of the company's research panel (*N* = 1680). Of these, 1120 completed the screening survey, and 640 eligible participants were invited to the main survey. The sample size was determined for the concurrent study [10], with reference to previously reported PSS utilization rates [3]. Although some variables were shared between the study [10] and current study, each has its own distinct purposes. Those who failed on the attention check were excluded by the company.

### 2.2 Measurements

#### 2.2.1 Screening Survey

Participants reported whether they were currently hospitalized, hospitalized for treatment or other reasons less than 5 days, or receiving outpatient care. They also reported the time since diagnosis. Participants who reported that five or more years had passed since the cancer diagnosis or not having been diagnosed with cancer were excluded from this survey.

#### 2.2.2 Main Survey

##### 2.2.2.1 Demographic Information

Participants reported their age, sex, current treatment status, cancer type, and physical functioning using the Japanese version of the Karnofsky Performance Scale [15].

##### 2.2.2.2 The Japanese Version of the Kessler Psychological Distress Scale (K6)

Psychological distress was assessed using the Japanese Version of K6, a six‐item screening scale [16]. The items were rated on a 5‐point scale (0–4). A cutoff score of 4/5 was used to indicate a possible mood or anxiety disorder [17]. Cronbach's *α* was 0.90.

##### 2.2.2.3 Brief Cancer‐Related Worry Inventory (BCWI)

Cancer‐related worry was measured using the BCWI [1]. This scale consists of 15 items rated on a 0–100 scale with 11 options including three subscales: future prospects, physical and symptomatic problems, and social and interpersonal problems. Cronbach's *α* was 0.94, 0.80, and 0.84, respectively.

##### 2.2.2.4 Use of Psychosocial Support Services

Participants reported whether they had ever used any PSS to cope with or resolve problems after diagnosis and, if not, whether they intended to use such services in the future. Eight response options were given to capture different stages of PSS use. Examples are: “I have used such services, but for less than 6 months.” and “I used such services in the past, but not currently.” Participants who currently or previously used PSS also reported the types of services they used (Supporting Information S1: Table S1).

##### 2.2.2.5 Reasons for Discontinuing or Terminating Psychosocial Support Services

Participants who had discontinued or terminated PSS selected all applicable reasons why they did so from the list that was developed based on previous studies (Table 1) [11, 12, 13, 18].

##### 2.2.2.6 Desire to Continue Using Psychosocial Support Services

Participants were asked if they were hoping to continue using PSS by selecting “yes”, “no”, or “neither”.

### 2.3 Statistical Analysis

Descriptive statistics were calculated. For the BCWI, mean scores for each subscale were computed. A one‐way analysis of variance (ANOVA) was used to examine the associations between desire to continue using PSS and psychological distress as well as cancer‐related worries. Welch's ANOVA was used when the assumption of homogeneity of variance was not met. All analyses were performed using IBM SPSS Statistics version 28.

### 2.4 Ethical Considerations

This study was approved by the Ethics Committee of the Graduate School of Human Sciences (Behavioral Sciences) at the University of Osaka (reference no. HB021‐138). Participants were informed about the study objectives on the cover page and provided informed consent prior to participation.

## 3 Results

### 3.1 Participants

A total of 510 participants provided valid responses (Supporting Information S1: Table S2). Of patients who discontinued or terminated PSS, most were breast cancer survivors, had been diagnosed 2–3 years earlier, and nearly 60% scored above the K6 cutoff.

### 3.2 Use of Psychosocial Support Services

Of 43 participants who had used PSS since diagnosis, 28 reported that they were not currently using PSS. The most frequently used services were cancer counseling and support centers (*n* = 14) (Supporting Information S1: Table S1). Current and past users reported higher levels in the K6 and all BCWI subscales than non‐users (all *p*s < 0.05) (Supporting Information S1: Table S3).

### 3.3 Reasons for Discontinuing or Terminating PSS (Table 1)

The most frequently reported reason for discontinuation/termination of PSS was *Needs fulfilled* (*n* = 15), which included responses such as “The problem had improved or been resolved.” *Enhanced coping resources* (*n* = 5) was also reported, including “I thought I would be able to handle the problems on my own.” *Structural/access barriers* (*n* = 4) included “I did not have enough time.” And, *Psychological burden* (*n* = 3) included “It felt burdensome to interact with others.” Six participants indicated *Service mismatch*, including “The services were different from what I had expected.” Responses provided in the “other” category were reviewed and categorized into the existing higher‐order categories. Frequencies of each category were calculated according to willingness to continue use and are presented in a table (Supporting Information S1: Table S4).

**TABLE 1:** Reasons for discontinuation/termination.

Reasons	*n*	%
***Needs fulfilled***	15	53.6
The problem had improved or been resolved.	12	42.9
I was satisfied with the services I received.	8	28.6
***Enhanced coping resources***	5	17.9
I thought I would be able to handle the problems on my own.	4	14.3
Support from family or friends was sufficient.	2	7.1
***Structural/access barriers***	4	14.3
The financial burden was substantial.	2	7.1
I did not have enough time.*	1	3.6
The service was not available in my area.*	1	3.6
The program was suspended due to the COVID‐19 pandemic.*	1	3.6
***Psychological burden***	3	10.7
It felt burdensome to interact with others.	3	10.7
***Service mismatch***	6	21.4
The services were different from what I had expected.	4	14.3
I did not think the services would improve my problems.	3	10.7
I was not sure what I was supposed to do there.	3	10.7

*Note:* Multiple responses were allowed. Items marked with an asterisk (*) came from open‐ended “Other” responses.

### 3.4 Psychological Distress and Cancer‐Related Worries by Willingness to Continue

We compared psychological distress and cancer‐related worries across the willingness to continue categories (Table 2). No significant differences were observed in the K6 or two domains of the BCWI. Patients who wished to continue tended to report higher levels of physical and symptomatic problems of the BCWI (*η* ^2^ = 0.127). Although the difference did not reach statistical significance in ANOVA and Levene's test was non‐significant (*p* = 0.063), this tendency was observed using Welch's ANOVA (*p* = 0.026).

**TABLE 2:** Results of one‐way ANOVAs for K6 and BWCI with willing to continue use.

	Yes		Neither		No		*F*(2, 25)	*p*	*η* ^2^
	(*n* = 6)		(*n* = 13)		(*n* = 9)				
	*M*	SD	*M*	SD	*M*	SD			
K6^a^	7.50	3.73	5.31	3.17	4.67	6.36	0.743	0.486	0.056
BCWI^b^									
Future prospects	64.72	20.07	61.80	20.87	53.52	24.89	0.566	0.575	0.043
Physical and symptomatic problems	60.83	8.47	46.73	28.01	37.22	22.31	1.824	0.182	0.127
Social and interpersonal problems	49.67	13.65	44.77	25.00	41.33	19.44	0.273	0.763	0.021

^a^ K6: The Japanese version of the Kessler Psychological Distress Scale.

^b^ BCWI: Brief Cancer‐Related Worry Inventory.

## 4 Discussion

This study focused on the post‐initiation process of HSB for PSS among patients with cancer. Discontinuation/termination of PSS was not limited to only negative aspects; rather, multiple reasons, both positive and negative, appeared to be involved.

### 4.1 Various Reasons Behind Discontinuation/Termination and Continued Use of Psychosocial Support Services

Various reasons were identified for why patients discontinued or terminated PSS. Importantly, such reasons included both negative and positive aspects, similar to previous reports [11, 12, 13]. The most common reason was *Needs fulfilled*, including improved or resolved problems, and satisfaction with the service. *Enhanced coping resources* suggested that some patients had or improved coping abilities or sufficient supports, being consistent with previous studies that certain types of PSS may promote problem‐solving skills and existing resources utilization [19, 20]. Although less frequently mentioned, *Structural/access barriers*, *Psychological burden*, and *Service mismatch* were also diverse and largely overlapped with previously identified barriers to PSS use [9]. Additionally, patients who wanted to continue using PSS tended to report situational or external reasons, whereas those who did not want to continue provided more positive explanations, as well as *Service mismatch* or *Psychological burden*.

Overall, the phenomenon often labeled as “dropout” may suggest that it encompasses qualitatively different experiences and processes. A similar perspective has been reported in research on psychological therapy for adolescents with depression [21]. According to a previous study [22], help‐seeking for emotional distress among patients with cancer reflects a process of “attaining normality.” From this perspective, seeking, accepting, or declining support can all function as adaptive responses depending on the individual's context and subjective appraisal. In this context, the discontinuation or termination of PSS use may reflect an ongoing, adaptive process in which patients evaluate the fit between their needs, available resources, and their desired sense of normality, rather than an indicator of service failure.

### 4.2 Examination of Needs After Discontinuation/Termination

Our findings showed that the levels of distress and worry among patients who have discontinued or terminated using PSS appeared comparable to those who are still using services. Notably, physical and symptomatic problems tended to be higher among patients who wished to continue. Therefore, even after discontinuation or termination, attention may be warranted for patients experiencing physical and symptomatic problems.

### 4.3 Implications

In developing systems for PSS delivery, it is important not only to prevent avoidable discontinuation or termination but also to explore patients' perspectives and facilitate reengagement with services as needed. Our results indicate that, for some patients, the discontinuation or termination of PSS may reflect desirable decisions, as it was due to problem resolution or enhanced coping abilities and resources. Medical staff involved in providing PSS should recognize the possibility of positive reasons and avoid uniformly labeling patients who discontinued as “dropouts.” Additionally, discontinuation may also reflect patients' attempts to regain or maintain a sense of normality [22]; thus, exploring each patient's normality may be an important support component for HSB. Moreover, given that some reasons for discontinuation/termination overlapped with previously identified barriers to PSS use, addressing these barriers may also contribute to promoting initial PSS use.

Among patients who discontinued or terminated PSS, those who wished to continue using the services tended to report higher levels of physical and symptomatic problems. Therefore, evaluations and follow‐up of PSS should consider the patients' needs regarding PSS use, levels of psychological distress, and the specific contents of worries. Proactive outreach strategies (e.g., telephone or email follow‐up) have been suggested to be beneficial for promoting PSS use [23]. Recommendations from medical staff to reengage in services at follow‐up doctor visits are also promising [7], particularly for patients who remain concerned about physical and symptomatic problems. Additionally, providing flexible options to reengage in PSS (e.g., eHealth interventions, lecture‐style programs) may be helpful for patients to overcome barriers to service use [14, 24].

### 4.4 Limitations

The current study adopted an online survey, thus, participants were limited to those with internet access and relatively high health awareness, which may have contributed to selection bias. However, this approach enabled recruitment beyond a single medical institution and allowed us to capture diverse types of PSS, including services that may not necessarily require continued use. Additionally, given the low utilization of PSS in Japan [3, 14], only 28 participants were classified as having discontinued or terminated services, which limited the statistical power to detect the significant differences. Third, participants classified as “discontinued/terminated users” may have included both planned completions and premature discontinuations, which could not be distinguished within the current study. Despite these limitations, our findings provide useful insights for the development of PSS delivery systems and for understanding HSB among patients with cancer.

## 5 Conclusion

Discontinuation or termination of PSS is not a uniform phenomenon; rather, it occurs due to both positive and negative reasons coexist. Moreover, those who reported wishing to continue services tended to be more worried about physical and symptomatic problems. These findings highlight the complexity of help‐seeking behavior and may inform the development of PSS delivery systems that support patients across different stages of service use.

## Author Contributions

Both authors contributed to the study planning and design. Tomoko Matsui was responsible for collecting data and conducting analyses. Tomoko Matsui prepared the first draft of the manuscript and Kanako Taku provided critical revisions and edited. Both authors have reviewed and approved the final manuscript.

## Funding

This work was supported by JSPS KAKENHI (Grant Number JP21K17667).

## Ethics Statement

This study was approved by the Ethics Committee of the Graduate School of Human Sciences (Behavioral Sciences) of the University of Osaka (reference number: HB021‐138).

## Consent

INTAGE Inc. explained the purpose of the online survey. Participants were considered to have provided their consent after responding to the survey.

## Conflicts of Interest

The authors declare no conflicts of interest.

## Supporting information


Supporting Information S1

## Data Availability

The datasets analyzed in this study are available from the corresponding author on reasonable request.
