# Supplementary material for: Reasons for Discontinuation or Termination of Help‐Seeking for Psychosocial Support Services Among Patients With Cancer in Japan
Source: Psychooncology. 2026 Nov 12;35(11):e70642. doi: 10.1002/pon.70642 (PMC13756558; doi:10.1002/pon.70642)
Supplement: Supplementary file 1 [file PON-35-e70642-s001.docx]

| Table S1 Psychosocial support services previously used by patients who discontinued or terminated use | | |  |
| --- | --- | --- | --- |
|  | *n* | *%* | |
| Cancer consulting and support center | 14 | 50.0 | |
| Psychiatry | 6 | 21.4 | |
| Regional medical cooperation office | 5 | 17.9 | |
| Medical consultation service | 4 | 14.3 | |
| Cancer salon | 4 | 14.3 | |
| Psychosomatic medicine | 3 | 10.7 | |
| Telephone consultation | 3 | 10.7 | |
| Counseling by therapist | 2 | 7.1 | |
| Psycho-oncology department | 1 | 3.6 | |
| Support group | 1 | 3.6 | |
| Supportive care team | 1 | 3.6 | |
| Palliative care team | 1 | 3.6 | |
| Email consultation | 1 | 3.6 | |
| Group therapy | 0 | 0 | |
| Others | 0 | 0 | |
| † Multiple responses were allowed. |  |  | |

Supporting Information

| Table S2 Demographic Information | | | |  |  |
| --- | --- | --- | --- | --- | --- |
|  | All (*n* = 510) | |  | Service discontinuation users (*n* = 28) | |
|  | *Mean* | *SD* |  | *Mean* | *SD* |
| *Age* | 62.1 | 11.8 |  | 54.5 | 9.3 |
|  | *n* | *%* |  | *n* | *%* |
| *Sex* |  |  |  |  |  |
| Male | 276 | 54.1 |  | 7 | 25.0 |
| Female | 234 | 45.9 |  | 21 | 75.0 |
| *Duration since diagnosis (months)* |  |  |  |  |  |
| Less than 1 year | 58 | 11.4 |  | 0 | 0 |
| 1–2years | 164 | 32.2 |  | 6 | 21.4 |
| 2–3years | 135 | 26.5 |  | 12 | 42.9 |
| 3–4years | 79 | 15.5 |  | 5 | 17.9 |
| 4–5years | 74 | 14.5 |  | 5 | 17.9 |
| *Cancer types* |  |  |  |  |  |
| Breast | 151 | 29.6 |  | 17 | 60.7 |
| Prostate | 87 | 17.1 |  | 0 | 0 |
| Colon | 67 | 13.1 |  | 2 | 7.1 |
| Lung | 39 | 7.6 |  | 2 | 7.1 |
| Stomach | 35 | 6.9 |  | 1 | 3.6 |
| Blood | 36 | 7.1 |  | 3 | 10.7 |
| Thyroid gland | 17 | 3.3 |  | 0 | 0 |
| Bladder | 16 | 3.1 |  | 0 | 0 |
| Uterine body | 12 | 2.4 |  | 1 | 3.6 |
| Pancreas | 12 | 2.4 |  | 0 | 0 |
| Liver | 10 | 2.0 |  | 0 | 0 |
| Cervical | 9 | 1.8 |  | 1 | 3.6 |
| Kidney | 8 | 1.6 |  | 1 | 3.6 |
| Esophagus | 7 | 1.4 |  | 1 | 3.6 |
| Ovarian | 6 | 1.2 |  | 0 | 0 |
| Brain | 4 | 0.8 |  | 1 | 3.6 |
| Others | 39 | 7.6 |  | 0 | 0 |
| *Treatment status (undergoing or within 6 months after treatment)* |  |  |  |  |  |
| Surgery | 29 | 5.7 |  | 0 | 0 |
| Chemotherapy | 96 | 18.8 |  | 3 | 10.7 |
| Radiation therapy | 21 | 4.1 |  | 1 | 3.6 |
| Hormone therapy | 161 | 31.6 |  | 11 | 39.3 |
| Others | 52 | 10.2 |  | 4 | 14.3 |
| *Karnofsky Performance Status* |  |  |  |  |  |
| Normal, no complaints | 252 | 49.4 |  | 9 | 32.1 |
| Able to carry on normal activities. Minor signs or symptoms of disease | 187 | 36.7 |  | 14 | 50.0 |
| Normal activity with effort | 49 | 9.6 |  | 3 | 10.7 |
| Care for self. Unable to carry on normal activity or to do active work | 17 | 3.3 |  | 2 | 7.1 |
| Requires occasional assistance, but able to care for most of his needs | 4 | 0.8 |  | 0 | 0 |
| Requires considerable assistance | 1 | 0.2 |  | 0 | 0 |
| Disabled, requires special care and assistance | 0 | 0 |  | 0 | 0 |
| Very sick, hospitalization is necessary | 0 | 0 |  | 0 | 0 |
| *K6 screening for psychological distress †* |  |  |  |  |  |
| Cutoff ≥5 | 156 | 30.6 |  | 16 | 57.1 |
| † K6 : The Japanese version of the Kessler Psychological Distress Scale | | | | | |

| Table 3 Results of One-way ANOVAs for K6 and BWCI with Use | | | | | | | | | | | | |
| --- | --- | --- | --- | --- | --- | --- | --- | --- | --- | --- | --- | --- |
|  | Current use | | | Discontinued/Terminated | |  | Never user | |  |  | |  |
|  | (*n* = 15) | |  | (*n* = 28) | |  | (*n* = 467) | |  |  | |  |
|  | *M* | *SD* |  | *M* | *SD* |  | *M* | *SD* |  | *F*(2, 507) | *p* | *η²* |
| K6 † | 8.20 | 6.22 |  | 5.57 | 4.49 |  | 3.32 | 4.16 |  | 12.815 | < 0.001 | 0.048 |
| BCWI ‡ |  |  |  |  |  |  |  |  |  |  |  |  |
| *Future prospects* | 60.00 | 21.59 |  | 59.76 | 21.73 |  | 40.68 | 25.68 |  | 11.232 | < 0.001 | 0.042 |
| *Physical and symptomatic problems* | 50.67 | 22.82 |  | 46.70 | 24.16 |  | 31.13 | 22.54 |  | 11.187 | < 0.001 | 0.042 |
| *Social and interpersonal problems* | 49.73 | 22.14 |  | 44.71 | 20.82 |  | 31.66 | 21.86 |  | 9.328 | < 0.001 | 0.035 |
| † K6 : The Japanese version of the Kessler Psychological Distress Scale | | | | | |  |  |  |  |  |  |  |
| ‡ BCWI : Brief Cancer-Related Worry Inventory | | |  |  |  |  |  |  |  |  |  |  |

| Table S4 Reasons and willing to continue | | | | | | |
| --- | --- | --- | --- | --- | --- | --- |
|  | Willing to continue use | | | | |  |
| Reasons | Yes（*n* = 6） | | Neither（*n* = 13） | | No（*n* = 9） | |
|  | *n* | *%* | *n* | *%* | *n* | *%* |
| Needs fulfilled | 2 | 33.3 | 8 | 61.5 | 5 | 55.6 |
| Enhanced coping resources | 0 | 0 | 4 | 30.8 | 1 | 11.1 |
| Structural/access barriers | 2 | 33.3 | 2 | 15.4 | 0 | 0.0 |
| Psychological burden | 1 | 16.7 | 1 | 7.7 | 1 | 11.1 |
| Service mismatch | 2 | 33.3 | 1 | 7.7 | 3 | 33.3 |
| † Multiple responses were allowed. | | | | | |  |
